# Autoantibodies to Ezrin are an early sign of pancreatic cancer in humans and in genetically engineered mouse models

**DOI:** 10.1186/1756-8722-6-67

**Published:** 2013-09-06

**Authors:** Michela Capello, Paola Cappello, Federica Caterina Linty, Roberto Chiarle, Isabella Sperduti, Anna Novarino, Paola Salacone, Giorgia Mandili, Alessio Naccarati, Carlotta Sacerdote, Stefania Beghelli, Samantha Bersani, Stefano Barbi, Claudio Bassi, Aldo Scarpa, Paola Nisticò, Mirella Giovarelli, Paolo Vineis, Michele Milella, Francesco Novelli

**Affiliations:** 1 Center for Experimental Research and Medical Studies (CeRMS), Azienda Ospedaliera Città della Salute e della Scienza di Torino, Turin, Italy; 2 Department of Molecular Biotechnology and Life Sciences, University of Torino, Turin, Italy; 3 Division of Biostatistics, Regina Elena National Cancer Institute, Rome, Italy; 4 Centro Oncologico Ematologico Subalpino (COES), Azienda Ospedaliera Città della Salute e della Scienza di Torino, Turin, Italy; 5 Gastroenterology Unit, Ordine Mauriziano Hospital, Turin, Italy; 6 Human Genetics Foundation, HuGeF, Turin, Italy; 7 Unit of Cancer Epidemiology, University of Turin and Center for Cancer Epidemiology and Prevention (CPO Piemonte), Turin, Italy; 8 ARC-NET Research Center, University of Verona, Verona, Italy; 9 Department of Pathology and Diagnostics, University of Verona, Verona, Italy; 10 Department of Surgery and Oncology, University of Verona, Verona, Italy; 11 Division of Immunology, Regina Elena National Cancer Institute, Rome, Italy; 12 Epigenetics Unit, Department of Surgery and Cancer, Imperial College, London, UK; 13 Division of Medical Oncology, Regina Elena National Cancer Institute, Rome, Italy

**Keywords:** Pancreatic ductal adenocarcinoma, Tumor antigen, Genetically engineered mouse model, Early diagnosis, Ezrin

## Abstract

**Background:**

Pancreatic Ductal Adenocarcinoma (PDAC) is a highly aggressive malignancy with only a 5% 5-year survival rate. Reliable biomarkers for early detection are still lacking. The goals of this study were (a) to identify early humoral responses in genetically engineered mice (GEM) spontaneously developing PDAC; and (b) to test their diagnostic/predictive value in newly diagnosed PDAC patients and in prediagnostic sera.

**Methods and results:**

The serum reactivity of GEM from inception to invasive cancer, and in resectable or advanced human PDAC was tested by two-dimensional electrophoresis Western blot against proteins from murine and human PDAC cell lines, respectively. A common mouse-to-human autoantibody signature, directed against six antigens identified by MALDI-TOF mass spectrometry, was determined. Of the six antigens, Ezrin displayed the highest frequency of autoantibodies in GEM with early disease and in PDAC patients with resectable disease. The diagnostic value of Ezrin-autoantibodies to discriminate PDAC from controls was further shown by ELISA and ROC analyses (*P* < 0.0001). This observation was confirmed in prediagnostic sera from the EPIC prospective study in patients who eventually developed PDAC (with a mean time lag of 61.2 months between blood drawing and PDAC diagnosis). A combination of Ezrin-autoantibodies with CA19.9 serum levels and phosphorylated α-Enolase autoantibodies showed an overall diagnostic accuracy of 0.96 ± 0.02.

**Conclusions:**

Autoantibodies against Ezrin are induced early in PDAC and their combination with other serological markers may provide a predictive and diagnostic signature.

## Background

Pancreatic ductal adenocarcinoma (PDAC) is the fourth leading cause of cancer death in Western countries. Upon diagnosis, less than 20% of patients present localized, potentially curable tumors. The overall 5-year survival rate is <5% [1,2]. This poor prognosis has been attributed to failure in early disease diagnosis, when the tumor may still be resectable, along with its propensity to disseminate and its resistance to systemic treatment [3]. CA19.9 is the only biomarker that has demonstrated clinical value for therapeutic monitoring and early detection of recurrent disease after treatment in patients with known pancreatic cancer. However, its use as a screening tool has proved unsuccessful, thus other biomarkers alone or in combination with it are required for early diagnosis of PDAC [1].

Autoantibody levels can function as diagnostic and prognostic markers [4,5]. By SERological Proteome Analysis (SERPA) we have previously identified a number of PDAC-associated antigens that are specifically recognized by circulating autoantibodies present in the serum of PDAC patients [6-9]. However as these autoantibodies were discovered in sera from patients at advanced stages of PDAC, earlier diagnostic markers would not have been identified.

Genetically engineered mice (GEM) that spontaneously develop PDAC may be used to facilitate the development of novel tests for the early detection and treatment of PDAC [10]. *LSL-Kras*^*G12D/+*^; *Pdx-1-Cre* mice (KC) develop the entire histologic compendium of pancreatic intraepithelial neoplasia (PanIN) lesions observed in the human disease, and a subset of mice also develop invasive pancreatic carcinomas. *LSL-Kras*^*G12D/+*^; *LSL-Trp53*^*R172H/+*^*; Pdx-1-Cre* double mutant mice (KPC), develop a more aggressive invasive and metastatic PDAC with an earlier time of onset, and display a reduced survival rate compared to KC mice [11,12].

In the present study, we used SERPA to identify TAAs eliciting an early humoral response in KC and KPC. Results from two-dimensional electrophoresis (2DE), Western blotting (WB) and mass spectrometry (MS) were combined to compare the reactivity of KC and KPC sera to that of corresponding matched controls. Antigens recognized by autoantibodies in KC and KPC at PanIN stages were identified and validated in a set of resectable and advanced PDAC patients. Ezrin (EZR), the protein with the highest frequency of autoantibodies in both early stage GEM and resectable PDAC patients, was validated by ELISA test using PDAC sera either collected at the time of diagnosis or several months before cancer onset (prediagnostic PDAC). The sensitivity and specificity of EZR-autoantibodies for discriminating PDAC was evaluated together with other serological markers.

## Methods

### Murine study

All animals were treated in accordance with European and institutional guidelines (Legislative Order No. 116/92). 129SvJae/B6 H-2D^b^ mice carrying mutated Kras^G12D^ and Trp53^R172H^ under the endogenous promoter, and flanked by Lox-STOP-Lox cassettes (*LSL-Kras*^*G12D/+*^ and *LSL-Trp53*^*R172H/+*^) were kindly provided from Dr. D.A. Tuveson (Cancer Research UK, Cambridge Research Institute, Cambridge, UK). C57BL/6 mice expressing Cre recombinase under a specific pancreatic transcriptional factor Pdx-1 (pancreatic duodenum homeobox 1) promoter *(Pdx-1-Cre*) were obtained from Dr. A.M. Lowy (University of California, San Diego, CA). Conditional *LSL-Kras*^*G12D/+*^, *LSL-Trp53*^*R172H/+*^ and *Pdx-1-Cre* strains were bred to obtain *LSL-Kras*^*G12D/+*^; *Pdx-1-Cre* single mutant (KC) and *LSL-Kras*^*G12D/+*^; *LSL-Trp53*^*R172H/+*^*; Pdx-1-Cre* double mutant (KPC) mice [11,12]. To collect serum, mice were euthanized and blood was collected by cardiac puncture using a 22-gauge needle and 1 ml syringe. Mice were surgically and pathologically examined to confirm the presence of pancreatic tumors and metastases.

### Human studies

#### ***Cross-sectional clinical study***

The study was approved by the Ethical Committees of: Azienda Ospedaliera Città della Salute e della Scienza di Torino, Turin; Policlinico G.B. Rossi, Verona; Regina Elena National Cancer Institute, Rome and Ordine Mauriziano Hospital, Turin. Serum samples were isolated from venous blood at time of diagnosis with the informed consent of patients and control subjects and stored at -80°C until use. De-identified numeric specimen codes were used to protect the identity of the individuals. Diagnosis of PDAC or any other cancer was consistently confirmed by histological or cytological analysis. Sera from 120 PDAC patients (M/F: 67/53; median age, 67 y; range, 32–86 y) with clinical features previously described [9] were analyzed by SERPA, and sera from 69 PDAC patients with clinical features described in Table 1 were tested by ELISA. Reactivity of these sera was compared, in both SERPA and ELISA studies, with that of control sera from the following sources: 60 healthy subjects (HS, M/F: 25/35; median age, 70 y; range, 49 - 90 y) with no prior history of cancer or autoimmune disease; 50 non-PDAC cancer patients (9 liver, 12 breast, 9 colon, 19 lung and 1 ovarian; M/F: 24/26; median age, 69 y; range, 44 - 86 y); 46 chronic pancreatitis patients (CP, M/F: 26/20; median age, 58 y; range, 22 - 74 y); 12 autoimmune diseases patients (AD, 3 Mixed Cryoglobulinemia, 2 Meniere's Syndrome, 4 Rheumatoid Arthritis, 2 Systemic Lupus Erythematosus, and 1 Autoimmune Pancreatitis; M/F: 3/9; median age, 49 y; range, 38 - 79 y).

**Table 1:** Clinical features of PDAC patients analyzed by ELISA

**Characteristics**			***N***	**%**^***a***^
**Gender**				
	Male		39	57
	Female		30	43
**Age (y)**				
	Mean	63	-	-
	Range	42-84	-	-
**Stage**^***b***^				
	IB		1	2
	IIA		7	10
	IIB		29	42
	III		10	14
	IV		22	32
**Grading**				
	Not reported		32	46
	1		4	6
	2		16	23
	3		17	25
**Primary site**				
	Head		49	71
	Body		6	9
	Tail		5	7
	Body-Tail		9	13
**ECOG PS**				
	Not reported		10	14
	0		32	47
	1		25	36
	≥2		2	3
**Surgery with radical intent**				
	Yes		39	57
	No		30	43
**Baseline CA19.9 (IU/ml)**				
	Evaluable		63	91
	Mean	3052	-	-
	Median	500	-	-
	Range	2- > 12000	-	-
**First-line chemotherapy**^***c***^				
	Evaluable		59	86
	Gem		43	73
	Gem/Oxal		10	17
	Gem/5-FU		3	5
	Non-Gem		1	2
	No CT		2	3
**ENOA1,2 Reactivity**				
	Evaluable		50	73
	Positive		34	68
	Negative		16	32

*ECOG PS* eastern cooperative oncology group performance status, *5-FU* 5-fluorouracil, *Gem* gemcitabine, *Oxal* oxaliplatin, *CT* chemotherapy.

^*a*^Rounded percentages.

^*b*^Classified according to the TMN classification of malignant tumor of the pancreas (UICC).

^*c*^First-line chemotherapy refers to palliative chemotherapy administered for relapsed, locally advanced inoperable, or metastatic disease.

#### ***Prospective pre-clinical study***

Prediagnostic serum samples of PDAC patients and matched controls were obtained from the Turin European Prospective Investigation into Cancer and Nutrition (EPIC) cohort that includes samples from 10 604 healthy subjects at the moment of enrolment (6 047 males and 4 557 females, aged 35–65 y) recruited in the city of Turin. Recruitment took place between 1993–1998 and involved blood donors and other healthy volunteers. After blood donation, samples were stored at 5–10°C, protected from light, and transported to local laboratories for processing and dividing into aliquots. Blood was separated into 0.5-ml fractions (serum, plasma, red cells, and buffy coat for DNA extraction) and stored in heat-sealed straws in liquid nitrogen (-196°C). Subjects were monitored longitudinally for cancer or other disease development. Co-operation with the local cancer registry and the local health authority enabled access to hospital discharge information and all newly diagnosed cancer cases. Study design, population and baseline data collection have previously been described in detail [13,14]. Sixteen PDAC patients identified from the Turin EPIC cohort are included in the present study. Controls were matched by age, sex, and date at entry in the cohort, and did not develop any cancer or autoimmune disease. Characteristics of subjects are summarized in Table 2. Each participant provided informed consent, and the local Ethics Review Committees approved this study.

**Table 2:** Characteristics of the EPIC cohort subjects

	**PDAC**		**Controls**	
	***N***	**%**	***N***	**%**
**Total**	16	100	32	100
**Age (y)**				
**Mean**	54.9		55.1	
**SD**	7.3		7.5	
**Sex**				
**Female**	7	44	14	44
**Male**	9	56	18	56
**Time span to diagnosis (mo)**				
**Mean**	61.2			
**Range**	5–117.1			

### Two-dimensional electrophoresis and western blot analysis

Cells (10^7^) from the CF-PAC-1 (ECACC ref. No. 91112501) and K8484 isolated from a tumor arising in KPC mice, kindly provided by Dr. K.P. Olive (Columbia University, New York, NY), were solubilized, subjected to 2DE and electro-transferred onto a nitrocellulose membrane (GE Healthcare Bio-Sciences, Uppsala, Sweden) as previously described [6]. Frozen PDAC tissues from eight surgically-treated patients (stage IIA and IIB of PDAC) were homogenized in 2DE lysis buffer, subjected to 2DE and electro-blotted onto a nitrocellulose membrane (GE Healthcare) as previously described [9]. Sera from KC, KPC, PDAC patients and controls were tested to determine mouse and human IgG concentrations using commercial kits (IgG ELISA Quantitation Set from Bethyl Laboratories - Montgomery, TX, USA). Sera were individually tested on 2DE maps at a working dilution of 0.1 mg/ml IgG for 4 h, followed by incubation with horseradish peroxidase (HRP)-conjugated rabbit anti-human IgG (90 minutes, 1:1000; Santa Cruz Biotechnology, Santa Cruz, CA, USA) or sheep anti-mouse Ig (90 minutes, 1:5000; GE Healthcare) as a secondary antibody. Ezrin spots were revealed with anti-Ezrin antibody (1 hour incubation, 1:5000; Abcam, Cambridge, MA, USA) and HRP-conjugated donkey anti-rabbit IgG (1 hour incubation, 1:2000; GE Healthcare) as a secondary antibody. Immunodetection was accomplished by ECL PLUS (Enhanced Chemiluminescence, GE Healthcare). The chemifluorescent signals were scanned with “ProXPRESS 2D” (PerkinElmer, Waltham, MA, USA) with an excitation/emission filter setting of 460/80 and 530/30, respectively, for an exposure time of 12 s. Images were recorded in TIFF format. The volume of each spot recognized by autoantibodies was calculated after background subtraction using “ProFinder 2D” (PerkinElmer) software and reported as arbitrary units (AU). For proteins represented from more than one spot the volume was expressed as a mean value.

### Protein identification by mass spectrometry

Coomassie G-stained spots were excised from 2DE preparative gels; destaining and in-gel enzymatic digestion were performed as previously described [15]. Briefly each spot was destained with 100 μl of 50% vol/vol acetonitrile in 5 mmol/l ammonium bicarbonate and dried with 100 μl of acetonitrile. Each dried gel piece was rehydrated for 40 minutes at 4°C in 10 μl of a digestion buffer containing 5 mmol/l ammonium bicarbonate, and 10 ng/μl of trypsin. Digestion was allowed to proceed overnight at 37°C and peptide mixtures were stored at 4°C until assayed. All digests were analyzed by a MALDI micro MX - TOF Mass Spectrometer (Waters, MA, USA) equipped with a delayed extraction unit. Peptide solution was prepared with equal volumes of saturated α-cyano-4-hydroxycinnamic acid solution in 40% vol/vol acetonitrile-0.1% vol/vol trifluoroacetic acid. The MALDI-TOF was calibrated with a mix of PEG (PEG 1000, 2000 and 3000 with the ratio 1:1:2) and mass spectra were acquired in the positive-ion mode. Peak lists were generated with ProteinLynx Data Preparation (ProteinLynx Global Server 2.2.5) using the following parameters: external calibration with lock mass using mass 2465.1989 Da of ACTH, background subtract type adaptive combining all scans, performing deisotoping with a threshold of 1%. The 25 most intense masses were used for database searches against the SWISSPROT database (Release 2011_12 of 14-Dec-11) using the free search program MASCOT 2.3.02 (http://www.matrixscience.com/cgi/search_form.pl?FORMVER=2&SEARCH=PMF). The following parameters were used in the searches: taxa *Homo sapiens* or *Mus musculus*, trypsin digest, one missed cleavage by trypsin, carbamidomethylation of cysteine as fixed modification, methionine oxidation as variable modifications and maximum error allowed 100 ppm. Only proteins with a Mascot score >55 were taken into consideration.

### Anti-Ezrin autoantibody capture by enzyme-linked immunosorbent assay

Purified recombinant protein of *Homo sapiens* Ezrin, transcript variant 1 (OriGene, Rockville, MD, USA) was used to capture autoantibodies to Ezrin. Briefly, the protein was coated (0.5 μg/ml in PBS) on 96-well micro-plates overnight at room temperature, followed by blocking with PBS containing 4% bovine serum albumin for 2 hours at room temperature. Sera (working dilution 0.01 mg/ml) were then added to the coated wells for 2 hours at room temperature. After washing with PBS-Tween-20, microplates were incubated with HRP-conjugated rabbit anti-human IgG (dilution 1:1000; Santa Cruz Biotechnology) for 1 hour at room temperature and TMB One Solution (Promega, Madison, WI, USA) was added to each well. The reaction was stopped by 2N HCl and the optical density (OD) value was measured at 450 nm. The corresponding background values of the sera on uncoated wells were subtracted. All samples were assayed in triplicate and the results represent mean values.

### Statistical analysis

Statistical analysis was performed using GraphPad (Version 4, San Diego, CA), MedCalc (Version 11.4.2.0, Mariakerke, Belgium) and SPSS (Version 18.0, Chicago, IL, USA) software packages. Mouse survival was estimated by Kaplan-Meier analysis and compared with Log-rank tests. Receiver operating characteristic (ROC) curve analysis was performed in order to find the optimal cut-off levels capable of splitting patients into groups with different outcome probabilities. Specificity, sensitivity and area under curve (AUC) were estimated considering histology results as the gold standard. The classification and regression tree (CART) analysis, a type of decision tree methodology, is a nonparametric statistical procedure that identifies mutually exclusive and exhaustive subgroups of a population whose members share common characteristics that influence the dependent variable of interest. CART uses a binary recursive partitioning method that produces a decision tree that identifies subgroups of patients with a higher likelihood of being found positive in a test for a disease state. The exhaustive CHAID method was used for CART analysis. Correlations and associations between variables were tested by Pearson’s test, Student's t-test, χ^2^ test or Fisher’s exact test, as appropriate. For all tests, 2-sided *P* < 0.05 (*), *P* < 0.005 (**) and *P* < 0.0005 (***) values were considered significant.

## Results

### Murine study

#### ***Serological proteome analysis in mice that spontaneously develop PDAC***

**Figure 1:**
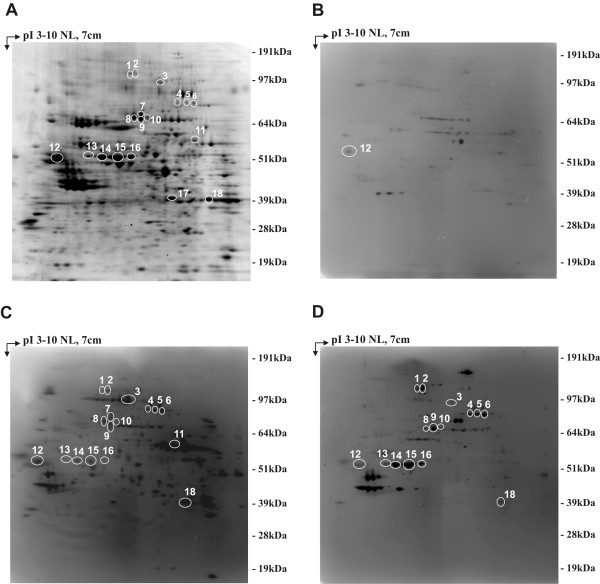
**SERPA analysis of KC and KPC serum reactivity against K8484 cell line 2DE map.** Total lysates from the K8484 cell line were separated by 2DE as described in the Methods section. Samples were focused in the first dimension using a gradient spanning the indicated pH range, separated in the second dimension in 4-12% acrylamide gels and subsequently Blue Coomassie stained **(A)** or transferred to a nitrocellulose membrane and probed with mouse sera. Three representative Western blot images show the immunoreactivity of control **(B)**, KC **(C)** or KPC **(D)** serum. Immunoreactive protein spots were determined for each serum by superimposition of immunoblot signal pattern with the spot pattern of the corresponding Blue Coomassie stained gel using the “ProFinder 2D” software. Numbered circles indicate immunoreactive proteins specifically recognized by KC and KPC sera and identified by MALDI-TOF MS. Immunoreactive protein names are listed in Additional file 1: Table S1.

**Figure 2:**
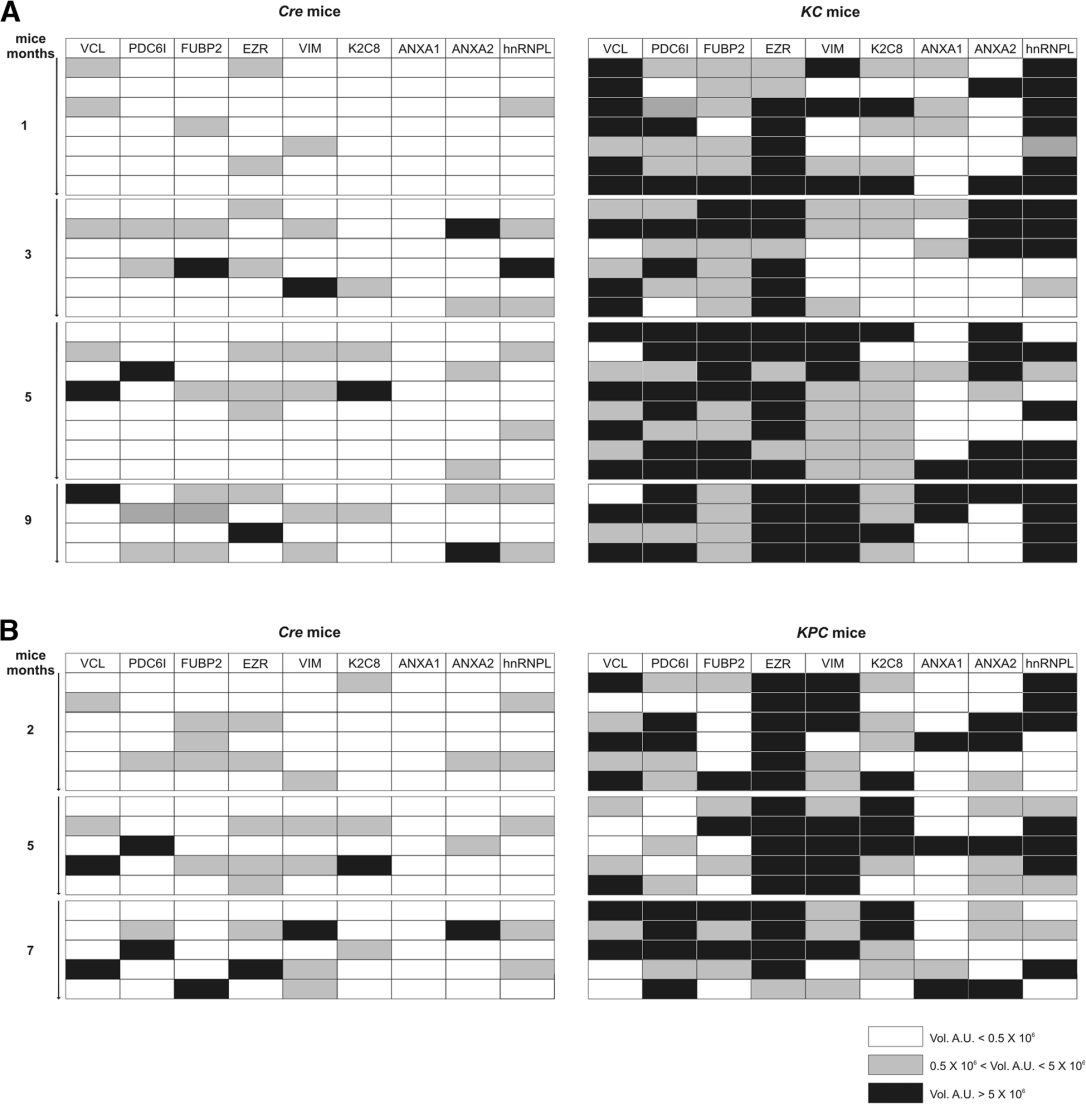
**Individual KC and KPC serum reactivity against the identified antigens.** The intensity of reactivity of each control Cre, KC **(A)** and KPC **(B)** serum against each MALDI-TOF MS identified protein is represented as a gray gradient scale of color as described in the legend. The volume (Vol) of each immunoreactive spot was calculated after background subtraction with the image analysis software “ProFinder 2D” and reported as arbitrary units (AU). For proteins represented from more than one spot the volume was expressed as mean AU value.

To identify tumor antigens associated with early PDAC development, we exploited two sophisticated mouse models of PDAC, KC and KPC mice, in which we could collect serum from inception of preinvasive disease to invasive cancer. KC and KPC displayed stereotypical neoplastic progression from pancreatic precursor lesions (PanIN) present at 1 month of age to advanced PDAC, showing a mean survival of 12 and 7 months, respectively (Additional file 1: Figure S1A). Percentages of transformed foci increased with age, ranging from less than 5% at 1 month of age to more than 80% at 9 and 5 months respectively (Additional file 1: Figure S1B-G). Serum samples collected from 25 KC at 1, 3, 5 and 9 months of age, and from 16 KPC at 2, 5 and 7 months of age were histologically attributed to different stages of tumor progression and subjected to SERPA. Total proteins extracted from the KPC-derived K8484 cell line were separated by 2DE and transferred onto nitrocellulose membranes. Sera from GEM or age-matched *Pdx-1-Cre* mice (hereafter defined as control mice) were screened individually for the presence of antibodies to PDAC proteins. Image analysis of the immunoreactivity identified 18 protein spots recognized at high frequency by KC and KPC sera compared to control mice sera (Figure 1A-D). These spots were excised from a preparative gel for MALDI-TOF analysis, leading to identification of nine proteins (Additional file 1: Table S1) belonging to three different functional groups, mainly cytoskeletal proteins or cytoskeleton regulators: Ezrin (EZR), Vimentin (VIM), Cytokeratin-8 (K2C8), Vinculin (VCL), Annexin A2 (ANXA2) and Annexin A1 (ANXA1); nuclear proteins: Far upstream element binding protein 2 (FUBP2) and Heterogeneous nuclear ribonucleoprotein L (hnRNPL) and a multifunctional protein: Programmed Cell Death-6 Interacting protein (PDC6I). The frequency of IgG to these proteins ranged between 0 and 44% for control mice, and between 25 and 100% for GEM sera (Figure 2A-B). Even the intensity of the reactivity of IgG to the identified proteins was significantly higher in GEM compared to matched controls (Figure 2A-B); 2DE WB performed with serum serial dilution displayed a higher titer of autoantibodies to these specific proteins in GEM compared to controls (data not shown). All antigens, and particularly EZR, VCL, VIM, PDC6I, hnRNPL and ANXA2 induced a specific antibody response in KC and KPC already at 1 to 3 months of age (Figure 2A-B), when the tumor stage was limited to early PanIN (Additional file 1: Figure S1B and E).

### Human studies

#### ***Serological proteome analysis in human PDAC***

To validate in PDAC patients the autoantibody signature identified in GEM, sera from 120 PDAC patients, 40 healthy subjects (HS), 50 non-PDAC tumor patients (non-PDAC), 46 chronic pancreatitis (CP) and 12 autoimmune disease (AD) patients, previously screened against α-enolase (ENOA) isoforms [9], were compared for reactivity against whole protein extracts of the CF-PAC-1 human PDAC cell line, resolved by 2DE. Only six antigens (EZR, ANXA2, VCL, hnRNPL, ANXA1 and PDC6I), represented by 12 spots, induced specific IgG in PDAC patients like in GEM with a frequency from 19% to 56% (Additional file 1: Figure S2 and Table S2 and Table 3).

**Table 3:** Frequencies of sera reactivity against protein spots in analyzed groups

**Spot no.**^**a**^	**Protein**	**PDAC**	**non-PDAC**	**CP**	**AD**	**HS**
		**(*N*** **= 120)**	**(*N*** **= 50)**	**(*N*** **= 46)**	**(*N*** **= 12)**	**(*N*** **= 40)**
**1-4**	**VCL**	31%	0%	9%	0%	5%
			*P* < 0.0001	*P* = 0.0024	*P* = 0.0193	*P* = 0.0005
**5**	**PDC6I**	21%	0 (0%)	7%	0%	3%
			*P* < 0.0001	*P* = 0.0361	*P* = 0.1221	*P* = 0.0033
**6-9**	**EZR**	56%	12%	2%	17%	10%
			*P* < 0.0001	*P* < 0.0001	*P* = 0.0135	*P* < 0.0001
**10**	**hnRNPL**	35%	4%	12%	0%	5%
			*P* < 0.0001	*P* = 0.0006	*P* = 0.0039	*P* < 0.0001
**11**	**ANXA1**	19%	0%	2%	0%	0%
			*P* = 0.0003	*P* = 0.0053	*P* = 0.2198	*P* = 0.0012
**12**	**ANXA2**	53%	8%	28%	0%	10%
			*P* < 0.0001	*P* = 0.0052	*P* = 0.0003	*P* < 0.0001

^*a*^Reactive protein spots numbered as shown in Additional file 1: Figure S2. Frequencies are expressed as percentage of positive sera; Fisher’s test was performed between PDAC and each control group, *P-*values < 0.05 were considered statistically significant.

**Figure 3:**
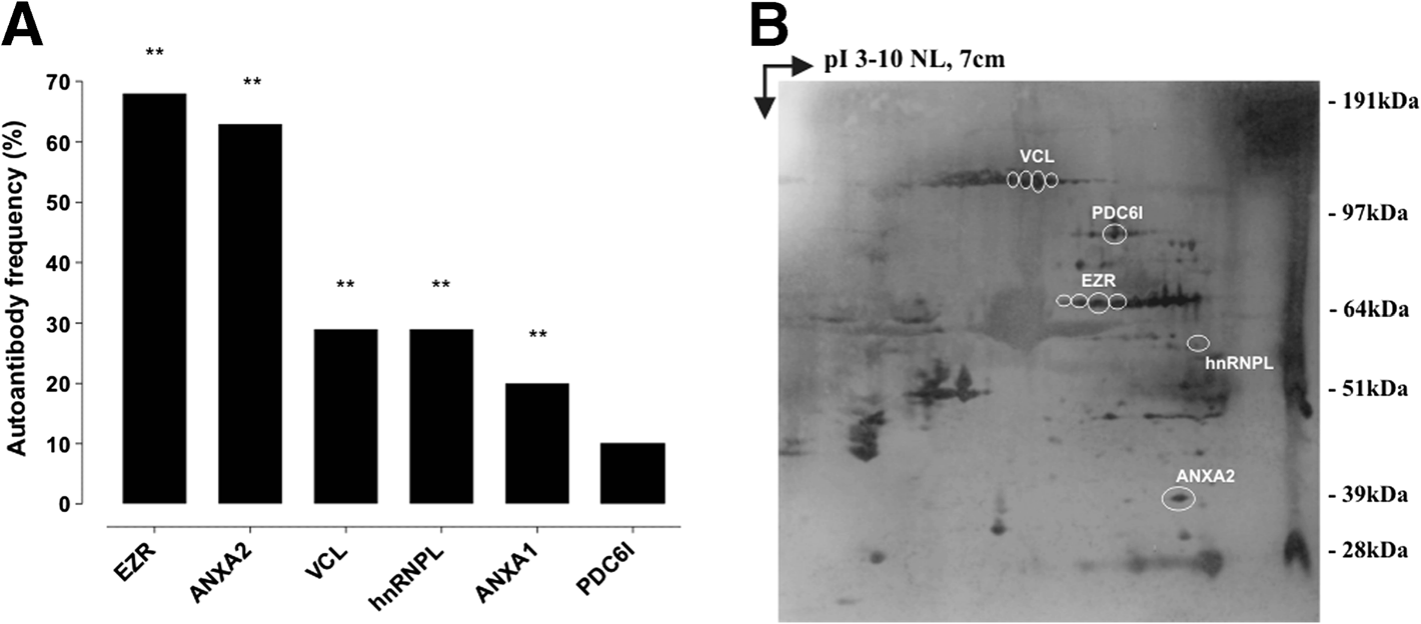
**Antigen validation in resectable PDAC patients. (A)** The graph shows the frequency of autoantibodies against mouse and human common immunoreactive antigens in the group of resectable patients who underwent surgery with curative intent (n = 38), analyzed by SERPA against CF-PAC-1 cell line 2DE map. P-values were calculated *vs*. control frequencies listed in Table 3 by Fisher's exact test (** P < 0.005). **(B)** Proteins were extracted from eight frozen PDAC tissues from surgically-treated patients (stage IIA and IIB), separated by 2DE, transferred to a nitrocellulose membrane and probed with the autologous serum. A representative Western blot is shown; circles indicate the presence of autoantibodies against the mouse and human common immunoreactive antigens.

IgG to these common antigens were not only present in advanced PDAC patients (*n* = 82), but also in the group of stage II and III resectable patients who underwent surgery with curative intent (*n* = 38). In this set of patients, there was a statistically significant frequency of autoantibodies against common antigens, with the exception of PDC6I, compared to controls; in particular, EZR and ANXA2 were recognized by 68 and 63% of resectable PDAC patients, respectively (Figure 3A). Moreover, for eight surgically-treated patients with stage IIA/IIB PDAC the antigen immunoreactivity was not only confirmed on the CF-PAC-1 cell lysate, but also on the autologous tumoral biopsy 2DE map (Figure 3B).

#### ***Cross-sectional clinical study: detection of anti-Ezrin autoantibodies***

EZR was the antigen recognized at the highest frequencies by sera from both GEM at early stages of the disease and resectable PDAC patients, and its identification was later proved through 2DE WB in K8484, CF-PAC-1 cells and PDAC tissues (Additional file 1: Figure 1). This analysis confirmed the presence of different EZR isoforms both in murine and human PDAC, likely due to different post-translational modifications (e.g. phosphorylation).

**Figure 4:**
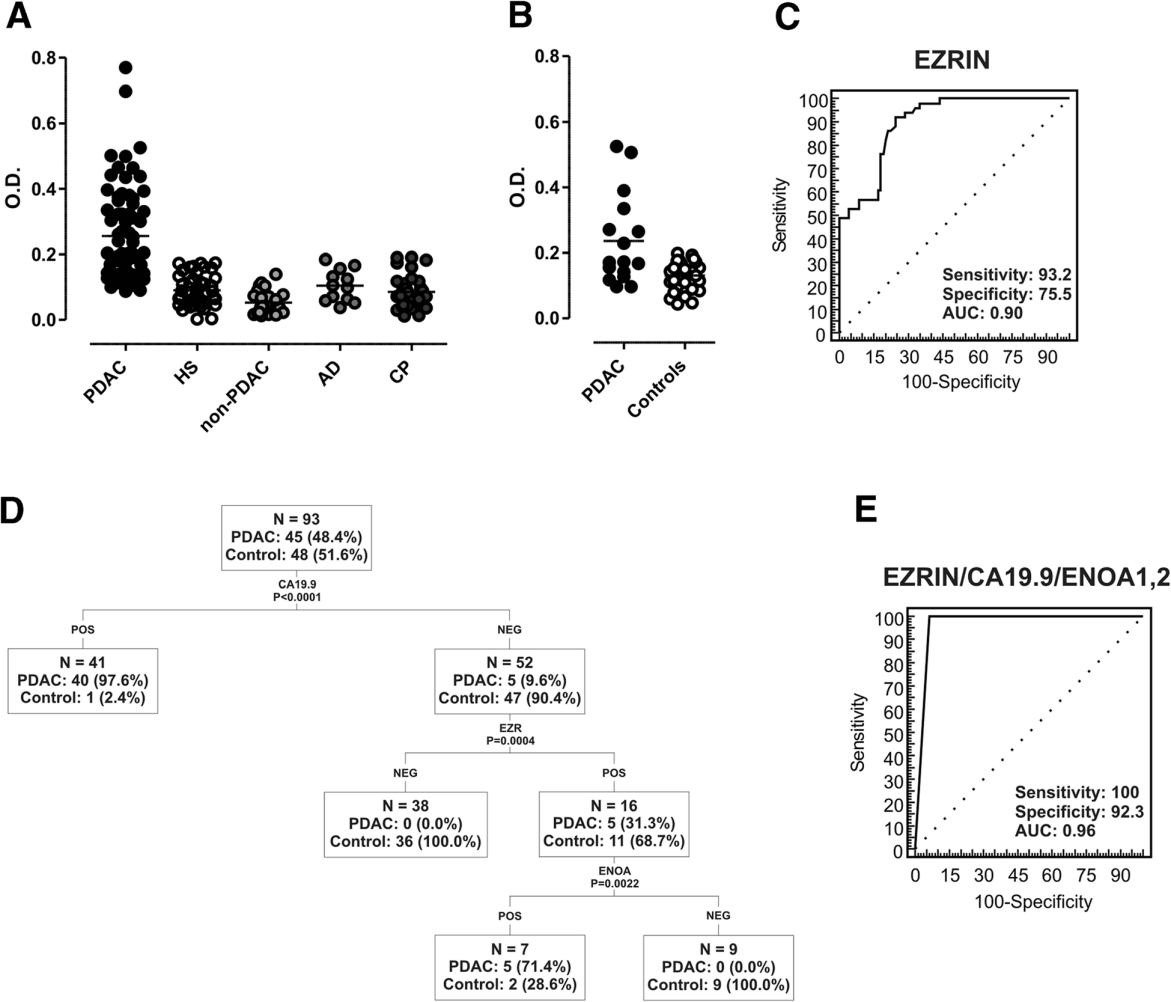
**Diagnostic performance of EZR-autoantibodies captured by ELISA. (A)** Scatter plots show the reactivity of PDAC (n = 69), healthy subject (HS, n = 45), non-PDAC (n = 28), autoimmune disease (AD, n = 12) and chronic pancreatitis (CP, n = 37) patient sera to EZR recombinant protein as assessed by ELISA: PDAC *vs*. HS, non-PDAC and CP P < 0.0001; PDAC *vs*. AD P = 0.0006. **(B)** Scatter plots show the reactivity of prediagnostic PDAC patient (n = 16) and matched control (n = 32) sera from the EPIC cohort to EZR recombinant protein as assessed by ELISA: PDAC *vs*. controls P = 0.0002. Reactivity is expressed as optical density (O.D.) read at 450 nm, P-values were calculated by Student's t-test. **(C)** ROC analysis of EZR-autoantibody sensitivity and specificity using O.D. obtained in ELISA as a continuous variable (cut-off value: O.D. = 0.1183). **(D)** Classification and regression tree (CART) analysis of CA19.9 serum levels (≥ 37 IU/ml), EZR-autoantibody reactivity (O.D. ≥ 0.1183) and ENOA1,2-autoantibody reactivity (expressed as 2DE WB positivity) with 93 PDAC patients and controls where all parameters were available. The number and percentage of PDAC patients and controls are shown for each node. **(E)** ROC analysis of sensitivity and specificity of EZR-autoantibody detection in combination with CA19.9 and ENOA1,2-autoantibodies in the cohort of samples where all three parameters were available (PDAC patients: n = 45; benign controls: HS, AD, CP, n = 48). The applied diagnostic algorithm assigns patients to the PDAC group when both EZR-autoantibodies and CA19.9 are positive, and separates discordant cases into PDAC or controls based on the presence or absence of ENOA1,2-autoantibodies.

Moreover, to approach the issue through a methodology with a wider clinical employment, we set up an ELISA test to validate 2DE WB results. The ability of sera from advanced (*n* = 30) and resectable (*n* = 39) PDAC patients (Table 1) to react against human recombinant EZR was compared to that of sera from HS (*n* = 45), non-PDAC tumor (*n* = 28; 8 breast, 8 colon, 12 lung cancer), CP (*n* = 37) and AD patients (*n* = 12). PDAC sera displayed specific reactivity to EZR protein (*P* < 0.0001, Figure 4A), but not to ANXA2, another protein recognized with a high frequency by resectable PDAC patients in 2DE WB (data not shown). The ELISA approach also confirmed the presence of autoantibodies against EZR in both KC and KPC, where there was a significant increase of autoantibody levels against recombinant EZR compared to control mice (data not shown).

#### ***Prospective pre-clinical study: validation of anti-Ezrin autoantibodies***

To investigate the occurrence of autoantibodies to EZR several months before PDAC diagnosis, we took advantage of the serum specimens from the European Prospective Investigation into Cancer and Nutrition (EPIC) cohort where blood samples were collected from healthy volunteers monitored longitudinally for cancer or other diseases development over the years. Sixteen prediagnostic PDAC patient specimens, with a time span to diagnosis of 5–117.1 mo (mean, 61.2 mo), and thirty-two matched controls from the Turin EPIC cohort were used for this study. Controls were matched for age, sex, and date of enrollment (Table 2). Notably, the level of autoantibodies to EZR was significantly higher in prediagnostic PDAC serum samples compared to matched controls (*P* = 0.0002), showing a similar trend of reactivity in both newly diagnosed and prediagnostic PDAC sera (Figure 4B). Detailed time lag to diagnosis and ELISA values for each PDAC patient of the EPIC cohort are shown in Additional file 1: Table S3.

### Analysis of diagnostic performance and clinical correlations

These ELISA results prompted us to use ROC analysis to assess the diagnostic performance of EZR-autoantibody detection. The analysis performed, using OD obtained in ELISA as a continuous variable, showed the greatest discriminating power between PDAC patients (*n* = 69) and benign controls (HS, CP and AD patients, *n* = 94) at a cut-off level of 0.1183 (sensitivity 93.2%, specificity 75.5%, AUC 0.90 ± 0.03; Figure 4C). With reference to the same cut-off, the ELISA test was also very efficient in discriminating between PDAC patients and patients with non-PDAC malignancies, with 94.9% sensitivity, 96.4% specificity, and an AUC of 0.99 ± 0.01 (data not shown). Thus we dichotomized the EZR-autoantibody variables into positive and negative according to the above-mentioned cut-off level (0.1183) for all subsequent analyses.

Dichotomized EZR-autoantibody levels did not show any correlation with clinical parameters (age, gender, stage at diagnosis, ECOG PS). However, mean and median OD values were significantly (*P* = 0.030) higher in PDAC patients who had undergone radical surgery (see also ROC analysis in Additional file 1: Figure S4, right panel).

We have previously demonstrated that autoantibodies to ENOA1,2, two phosphorylated isoforms of ENOA, usefully complement the diagnostic performance of CA19.9 serum levels [9,16]. Thus, we tested by CART analysis the diagnostic performance of EZR-autoantibodies in concomitance with the above biomarkers: CA19.9 (using the most relevant clinical laboratory cut-off value of 37 IU/ml) and ENOA1,2-autoantibodies, in the cohort of samples where all three parameters were available (PDAC patients: n = 45; HS, AD, CP: n = 48) (Figure 4D). Using this approach, the first node that significantly discriminated PDAC patients from benign controls was CA19.9 (*P* < 0.001). EZR-autoantibodies significantly refined CA19.9 diagnostic performance, particularly in CA19.9-negative cases (*P* < 0.0001). Finally, in the few cases in which CA19.9 and EZR-autoantibodies yielded conflicting results (i.e. CA19.9 negative/EZR-autoantibodies positive), ENOA1,2-autoantibodies significantly improved diagnostic performance (*P* = 0.0022). Since only one control subject in our cohort displayed high CA19.9 serum levels, CART analysis could not further classify CA19.9 positive cases. However, among CA19.9 positive cases only one PDAC patient and the above mentioned control subject resulted negative for EZR-autoantibodies, and they were again properly classified by ENOA1,2-autoantibodies. Based on these findings, a diagnostic algorithm which assigned patients to the PDAC group when both EZR-autoantibodies and CA19.9 were positive, and separated discordant cases into PDAC or controls based on the presence or absence of ENOA1,2-autoantibodies, respectively, resulted in 100% sensitivity and 92.3% specificity, with an overall diagnostic accuracy (AUC) of 0.96 ± 0.02 (Figure 4E). The diagnostic performance of EZR-autoantibody, CA19.9 and ENOA1,2-autoantibody tested individually in the same cohort is shown in Additional file 1: Figure S4.

While EZR autoantibodies have diagnostic potential, they did not show the prognostic value we have previously reported for ENOA1,2 [9]. Even though PDAC patients who had experienced disease control (either partial response or stable disease) upon first-line chemotherapy had significantly (*P* = 0.030) higher mean and median OD values in the EZR ELISA, EZR-autoantibodies analyzed as a continuous variable had no significant impact on survival (PFS or OS), either following surgery with radical intent or first-line chemotherapy. This demonstrates the power of the SERPA approach, combined with ELISA, to identify antigens that could serve distinct functions in an armament of tools for the diagnosis and prognosis of PDAC.

## Discussion

This study identifies autoantibodies to EZR as early markers in mouse and human PDAC. Of clinical relevance, we also show that EZR-autoantibodies efficiently complement the diagnostic performance of CA19.9.

To identify early immune response markers we applied SERological Proteome Analysis (SERPA) in KC and KPC mice spontaneously developing PDAC. As GEM can be sampled at defined stages of tumor development and under controlled breeding conditions, greater standardization is possible when using mouse models as opposed to human studies. GEM allowed us to identify EZR-autoantibodies as early biomarkers in PDAC, since precociously detected in their serum when the disease stage was limited to PanIN. Through this approach, we also identified additional antigens (VCL, PDC6I, FUBP2, hnRNPL, VIM, K2C8, ANXA1 e ANXA2) recognized at high frequencies by both KC and KPC sera.

Reactivity against some of these antigens was present in control mice, but the intensity of WB recognition was much greater in GEM. A clear example is represented by EZR, faintly recognized by a number of control mice but strongly evident in all KPC. Despite the fact that auto-reactive lymphocytes should have been removed from the repertoire before maturation into naïve B cells, a large number of circulating IgG^+^ memory B cells produce low affinity antibodies to self-antigens [17,18]. The humoral response against these self-antigens is strongly increased in tumor conditions, as demonstrated in this work both in humans and in mice (Figure 1, Table 3 and Figure 4). Some differences in the pattern of recognition were present between mice of the same age, probably due to the molecular heterogeneity of tumor progression in this model that fully recapitulates the genetic and molecular features of human PDAC [12]. Importantly, all the identified antigens, except for FUBP2 and ANXA1, induced a powerful humoral response not only in KPC but also in KC bearing PanIN lesions, indicating that the antibody response to these TAAs is already occurring when *Kras* is the only genetic alteration in the tumor, independently of *p53* mutation, which is a later event in PDAC development. This reflects previous studies reporting how the immune response to TAAs in humans occurs at an early stage during tumorigenesis, as illustrated by the detection of high titers of autoantibodies, as early as 5 years before disease onset [19,20].

By comparing the 2DE WB reactivity of GEM with that of a large cohort of PDAC patients and controls, six proteins, namely: EZR, ANXA2, VCL, hnRNPL, ANXA1 and PDC6I were common to both human and mouse signatures. EZR and ANXA2 were recognized by most PDAC patients who underwent surgery with curative intent. ELISA confirmed the diagnostic value of anti-EZR but not anti-ANXA2 autoantibodies, which were also present in control groups. Other studies have indeed reported the presence of autoantibodies against ANXA2 in systemic autoimmune diseases and lung cancer, as well as pancreatic cancer [21-23], suggesting that the humoral response to ANXA2 is not specific for PDAC transformation.

EZR is a member of the ezrin-radixin-moesin (ERM) family and a link between a number of growth factor receptors/adhesion molecules and the actin cytoskeleton. It is localized to the cytoplasm as an inactive form. Upon threonine and tyrosine phosphorylation, EZR is transported to the cell membrane whereupon it tethers F-actin [24]. It works downstream of cell-surface receptors through the activation of Rho and PI3K/Akt signaling pathways [25,26], and in physiological conditions, EZR is required for macropinocytosis, cell adhesion, and membrane ruffling in epithelial cells, whereas in tumor cells it is an important metastatic regulator [27]. EZR is overexpressed in many cancers, including PDAC, even in PanIN lesions [28-30], and it interacts with cortactin to form podosomal rosettes in PDAC cells, which may play an important role in tumor invasion [31]. These observations support the immunogenicity of EZR that we observed in the present study, even if it is not clear how TAAs overcome self-tolerance and thus become autoantibody targets in cancer patients, as many of those discovered so far are intracellular proteins [4,32,33]. Interestingly, EZR has been identified both in exosomes secreted by mesothelioma cells [34] and as a substrate of matrix metalloproteinases able to generate neo-epitopes from self-antigens [35].

The most important observation of our study is that autoantibodies against EZR were present also in prediagnostic PDAC samples from the prospective EPIC cohort that were collected several months or years before PDAC diagnosis. The EPIC study recruited over half a million healthy volunteers in ten European countries, including Italy, monitored longitudinally for cancer or other disease development [36]. Since it has been estimated that the elapsed time between PDAC initiation to metastatic spread is at least 10 years [37], our results strongly support the hypothesis that EZR-autoantibody development is an early event in PDAC. Notably, prediagnostic patients with the highest levels of EZR-autoantibody in the ELISA test were the ones with an intermediate time lag to diagnosis (69.3 and 56.9 mo, Additional file 1: Table S3). This observation supports the hypothesis that autoantibody levels decrease closer to diagnosis due to immune complex formation [20].

Although EZR-autoantibody testing has displayed a high diagnostic performance, especially in resectable PDAC patients (Additional file 1: Figure S4), a single TAA may lack adequate sensitivity and specificity, and the combination of a panel of autoantibodies and serological markers can improve the overall accuracy of a diagnostic assay for cancer detection. We therefore assessed the diagnostic performance of combined dichotomized EZR-autoantibody levels, CA19.9, the only PDAC marker currently in clinical use, and ENOA1,2-autoantibodies. We have previously demonstrated that autoantibodies against Ser-419-phosphorylated ENOA isoforms (ENOA1,2) complement the performance of CA.19.9 [9]. Interestingly, a diagnostic algorithm separating CA19.9 and EZR-autoantibodies discordant cases into PDAC or controls based on the presence or absence of ENOA1,2-autoantibodies respectively, resulted in an overall diagnostic accuracy of 0.96. Notably, the algorithm here applied is more stringent than the one previously described by our group [9], where tested cases were assigned to the PDAC group when either ENOA1,2-autoantibodies or CA19.9 were positive, as their values were inversely correlated. This finding is of real translational relevance, since CA19.9 is the only biomarker with demonstrated clinical value for therapeutic monitoring and detection of recurrent PDAC, but its use as a screening tool has proved unsuccessful until now [3].

Further validation studies, performed in a large and independent patient cohort, are warranted to establish the diagnostic performance of this multiplexed analysis and of the identified TAA panel tested alone or in combination.

## Abbreviations

2DE: Two dimensional electrophoresis; AD: Autoimmune disease; ANXA1: Annexin A1; ANXA2: Annexin A2; AU: Arbitrary units; AUC: Area under the curve; CART: Classification and regression tree; CP: Chronic pancreatitis; ECOG PS: Eastern cooperative oncology group performance status; ELISA: Enzyme-linked immunosorbent assay; ENOA: α-enolase; EPIC: European Prospective Investigation into Cancer and Nutrition; ERM: Ezrin-radixin-moesin; EZR: Ezrin; FUBP2: Far upstream element binding protein 2; GEM: Genetically engineered mice; hnRNPL: Heterogeneous nuclear ribonucleoprotein L; HRP: Horseradish peroxidase; HS: Healthy subject; K2C8: Cytokeratin-8; KC: *LSL-Kras*^*G12D/+*^, *Pdx-1-Cre* mice; KPC: *LSL-Kras*^*G12D/+*^, *LSL-Trp53*^*R172H/+*^*, Pdx-1-Cre* mice; MALDI-TOF: Matrix-assisted laser desorption ionization-time of flight; MS: Mass spectrometry; non-PDAC: Non-pancreatic cancer; NP: Normal pancreas; OD: Optical density; PanIN: Pancreatic intraepithelial neoplasia; PDAC: Pancreatic ductal adenocarcinoma; PDC6I: Programmed cell death-6 interacting protein; Pdx-1: Pancreatic duodenum homeobox 1; ROC: Receiver operating curve; SEREX: Serological analysis of tumor antigens by recombinant cDNA expression cloning; SERPA: Serological proteome analysis; TAA: Tumor-associated antigen; VCL: Vinculin; VIM: Vimentin; WB: Western blot.

## Competing interests

FN, MC, and PC are inventors of an Italian patent application No: TO2012A000523 entitled “Kit for *in vitro* diagnosis and predisposition assessment of pancreatic ductal adenocarcinoma”. Potential investigator conflict of interest has been disclosed to study participants.

## Authors’ contributions

MC designed the study, performed human SERPA and ELISA experiments, analyzed the data and wrote the manuscript; PC designed the study, coordinated and performed GEM breeding, murine sample collection and analyzed data; FCL performed murine SERPA studies and GEM serum collection; MG contributed to GEM breeding and analyzed data; RC performed GEM histological and immunohistochemical analysis; IS performed statistical analysis; GM performed mass spectrometry analysis; SB and SB performed human histological and immunohistochemical analysis; SB performed microarray analysis; AN, PN, PS, AS, CB and MM recruited patients and contributed to experimental design and analysis of data; AN, CS and PV provided samples from the Turin EPIC cohort and analyzed data; FN supervised the project and wrote the manuscript. All authors read and approved the final manuscript.

## Supplementary Material

Additional file 1: Figure S1 Survival curve and histological progression of KC and KPC. **Table S1.** Identification of proteins recognized by GEM sera using MALDI-TOF MS. **Table S2.** Identification of proteins recognized by PDAC patient sera using MALDI-TOF MS. **Figure S2.** Immunoreactivity of PDAC patient and control sera against CF-PAC-1 cell line 2DE map. **Figure S3.** Validation of EZR identification by Western blot analysis. **Table S3.** Time span to diagnosis and EZR-autoantibody ELISA values of PDAC patients from the EPIC cohort. **Figure S4.** ROC analysis of individually evaluated EZR-autoantibody, dichotomized CA19.9 serum level and ENOA1,2-autoantibody. Click here for file
