# Supplementary material for: Autoantibodies to Ezrin are an early sign of pancreatic cancer in humans and in genetically engineered mouse models
Source: J Hematol Oncol. 2013 Sep 6;6:67. doi: 10.1186/1756-8722-6-67 (PMC3844582; doi:10.1186/1756-8722-6-67)
Supplement: Additional file 1: Figure S1 — Survival curve and histological progression of KC and KPC. Table S1. Identification of proteins recognized by GEM sera using MALDI-TOF MS. Table S2. Identification of proteins recognized by PDAC patient sera using MALDI-TOF MS. Figure S2. Immunoreactivity of PDAC patient and control sera against CF-PAC-1 cell line 2DE map. Figure S3. Validation of EZR identification by Western blot analysis. Table S3. Time span to diagnosis and EZR-autoantibody ELISA values of PDAC patients from the EPIC cohort. Figure S4. ROC analysis of individually evaluated EZR-autoantibody, dichotomized CA19.9 serum level and ENOA1,2-autoantibody. [file 1756-8722-6-67-S1.doc]

**Supplementary Figures And Tables**


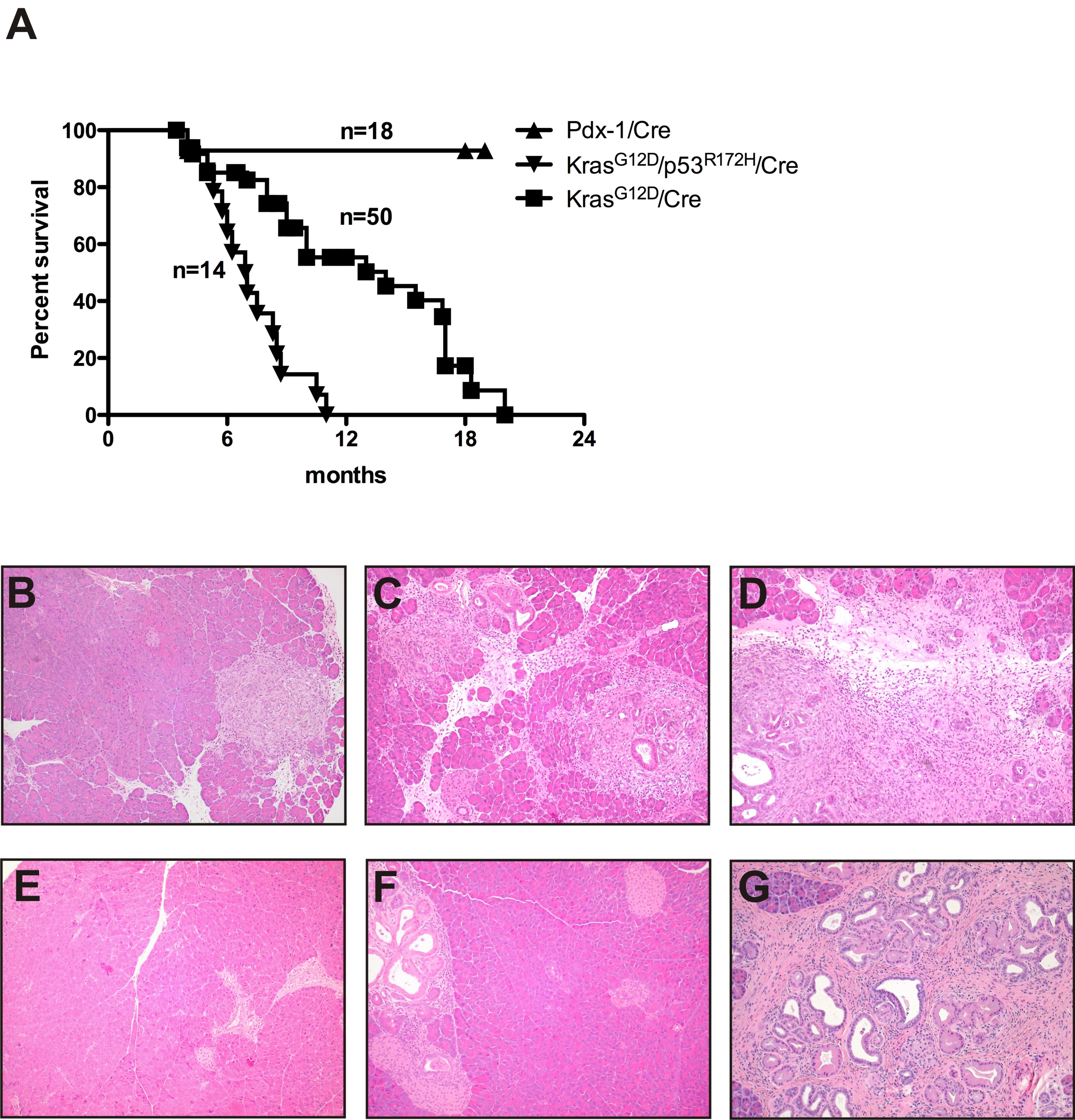


**Figure S1 Survival curve and histological progression of KC and KPC.**

**Table S1** **Identification of proteins recognized by GEM sera using MALDI-TOF MS**

| **Spot no.^a^** | **Accession no.^b^** | **Protein name** | **pI** | **MW (kDa)** | **Queries matched** | **Coverage (%)** | **Score** |
| --- | --- | --- | --- | --- | --- | --- | --- |
| 1 | Q64727 | Vinculin (VCL) | 5.8 | 117.2 | 18 | 19% | 158 |
| 2 | Q64727 | Vinculin (VCL) | 5.8 | 117.2 | 17 | 19% | 147 |
| 3 | Q9WU78 | Programmed Cell Death-6 Interacting protein (PDC6I) | 6.2 | 96.5 | 9 | 14% | 73 |
| 4 | Q3U0V1 | Far upstream element binding protein 2 (FUBP2) | 6.9 | 77.2 | 9 | 19% | 89 |
| 5 | Q3U0V1 | Far upstream element binding protein 2 (FUBP2) | 6.9 | 77.2 | 15 | 26% | 167 |
| 6 | Q3U0V1 | Far upstream element binding protein 2 (FUBP2) | 6.9 | 77.2 | 13 | 20% | 132 |
| 7 | P26040 | Ezrin (EZR) | 6.2 | 69.4 | 16 | 24% | 128 |
| 8 | P26040 | Ezrin (EZR) | 6.2 | 69.4 | 18 | 29% | 170 |
| 9 | P26040 | Ezrin (EZR) | 6.2 | 69.4 | 21 | 33% | 221 |
| 10 | P26040 | Ezrin (EZR) | 6.2 | 69.4 | 21 | 33% | 221 |
| 11 | Q8R081 | Heterogeneous nuclear ribonucleoprotein L (hnRNPL) | 8.3 | 63.9 | 15 | 28% | 140 |
| 12 | P20152 | Vimentin (VIM) | 4.9 | 53.7 | 14 | 43% | 158 |
| 13 | P11679 | Keratin, type II cytoskeletal 8 (K2C8) | 5.7 | 54.6 | 16 | 37% | 175 |
| 14 | P11679 | Keratin, type II cytoskeletal 8 (K2C8) | 5.7 | 54.6 | 16 | 38% | 166 |
| 15 | P11679 | Keratin, type II cytoskeletal 8 (K2C8) | 5.7 | 54.6 | 19 | 37% | 208 |
| 16 | P11679 | Keratin, type II cytoskeletal 8 (K2C8) | 5.7 | 54.6 | 17 | 35% | 196 |
| 17 | P10107 | Annexin A1 (ANXA1) | 7.0 | 38.9 | 14 | 54% | 176 |
| 18 | P07356 | Annexin A2 (ANXA2) | 7.6 | 38.9 | 16 | 47% | 213 |

^a^Spots numbers as shown in Figure 1; ^b^Accession number according to UniProtKB Database.

**Table S2 Identification of proteins recognized by PDAC patient sera using MALDI-TOF MS**

| **Spot no.^a^** | **Accession no.^b^** | **Protein name** | **pI** | **MW(kDa)** | **Queries matched** | **Coverage (%)** | **Score** |
| --- | --- | --- | --- | --- | --- | --- | --- |
| 1 | P18206 | Vinculin (VCL) | 5.8 | 117.2 | 12 | 14% | 83 |
| 2 | P18206 | Vinculin (VCL) | 5.8 | 117.2 | 18 | 20% | 176 |
| 3 | P18206 | Vinculin (VCL) | 5.8 | 117.2 | 14 | 16% | 114 |
| 4 | P18206 | Vinculin (VCL) | 5.8 | 117.2 | 19 | 21% | 181 |
| 5 | Q8WUM4 | Programmed Cell Death-6 Interacting protein (PDC6I) | 6.1 | 96.0 | 11 | 19% | 109 |
| 6 | P15311 | Ezrin (EZR) | 5.9 | 69.4 | 15 | 28% | 130 |
| 7 | P15311 | Ezrin (EZR) | 5.9 | 69.4 | 14 | 24% | 119 |
| 8 | P15311 | Ezrin (EZR) | 5.9 | 69.4 | 10 | 16% | 86 |
| 9 | P15311 | Ezrin (EZR) | 5.9 | 69.4 | 13 | 17% | 97 |
| 10 | P14866 | Heterogeneous nuclear ribonucleoprotein L (hnRNPL) | 8.4 | 64.1 | 15 | 23% | 128 |
| 11 | P04083 | Annexin A1 (ANXA1) | 6.6 | 38.7 | 13 | 46% | 166 |
| 12 | P07355 | Annexin A2 (ANXA2) | 8.5 | 40.7 | 11 | 40% | 141 |

^a^ Number of spots are referred to Figure S2; ^b^Accession number according to UniProtKB Database.

**
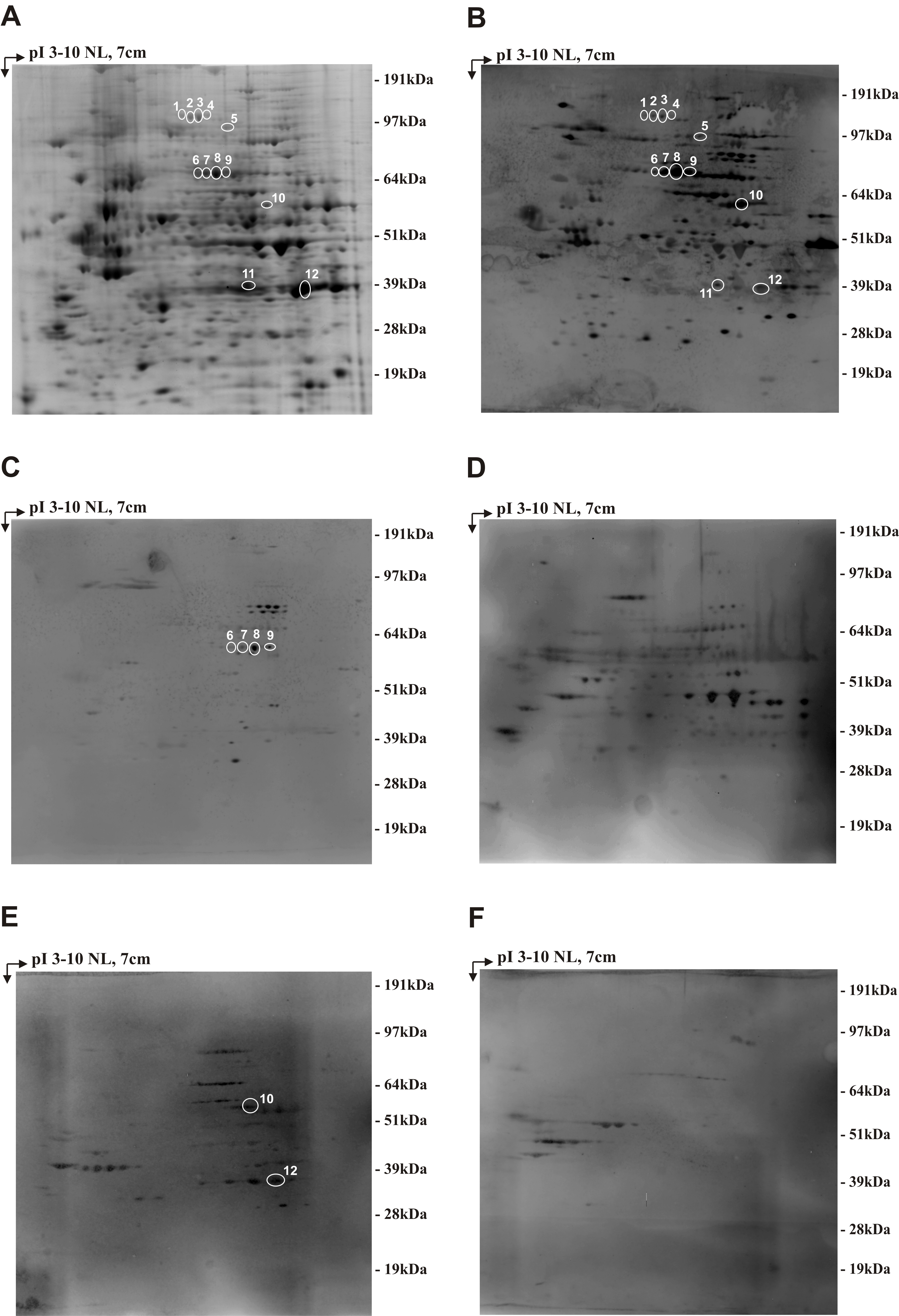
**

**Figure S2 Immunoreactivity of PDAC patient and control sera against CF-PAC-1 cell line 2DE map**.

**
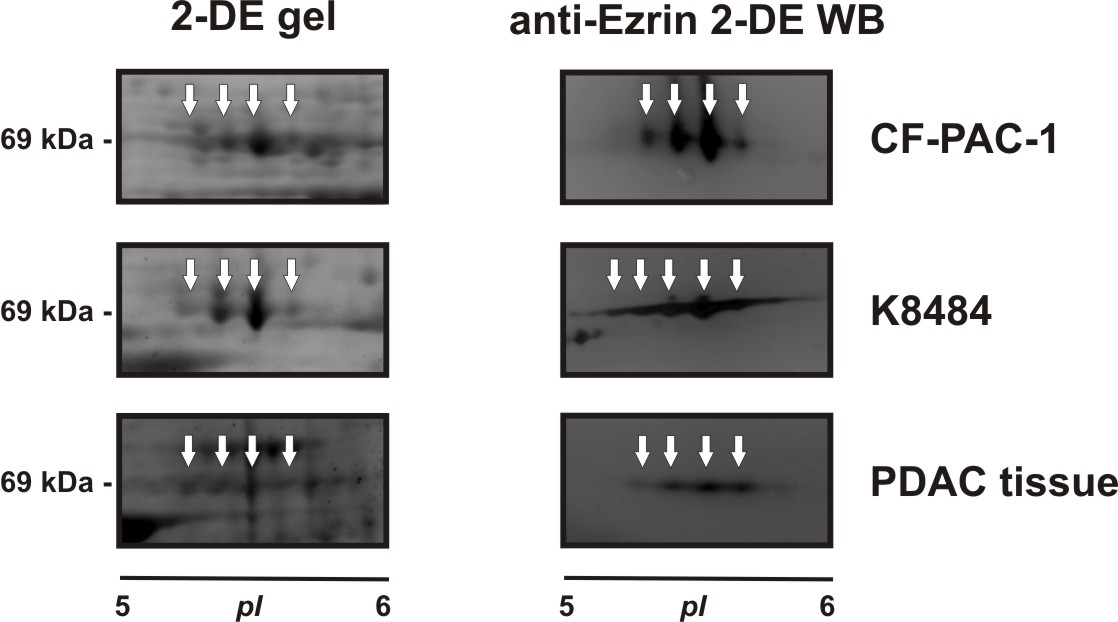
**

**Figure S3 Validation of EZR identification by Western blot analysis.**

**Table S3 Time span to diagnosis and EZR-autoantibody ELISA values of PDAC patients from the EPIC cohort**

| **Patient no.** | **Time span to diagnosis (mo)** | **ELISA O.D. value** |
| --- | --- | --- |
| 1 | 117.1 | 0.128 |
| 2 | 113.3 | 0.156 |
| 3 | 103.9 | 0.173 |
| 4 | 96.4 | 0.168 |
| 5 | 85.6 | 0.271 |
| 6 | 77.1 | 0.097 |
| 7 | 75.1 | 0.265 |
| 8 | 69.3 | 0.525 |
| 9 | 56.9 | 0.507 |
| 10 | 51.3 | 0.097 |
| 11 | 45.6 | 0.118 |
| 12 | 39.5 | 0.229 |
| 13 | 22.1 | 0.390 |
| 14 | 14.9 | 0.143 |
| 15 | 6.2 | 0.335 |
| 16 | 5.0 | 0.172 |

**
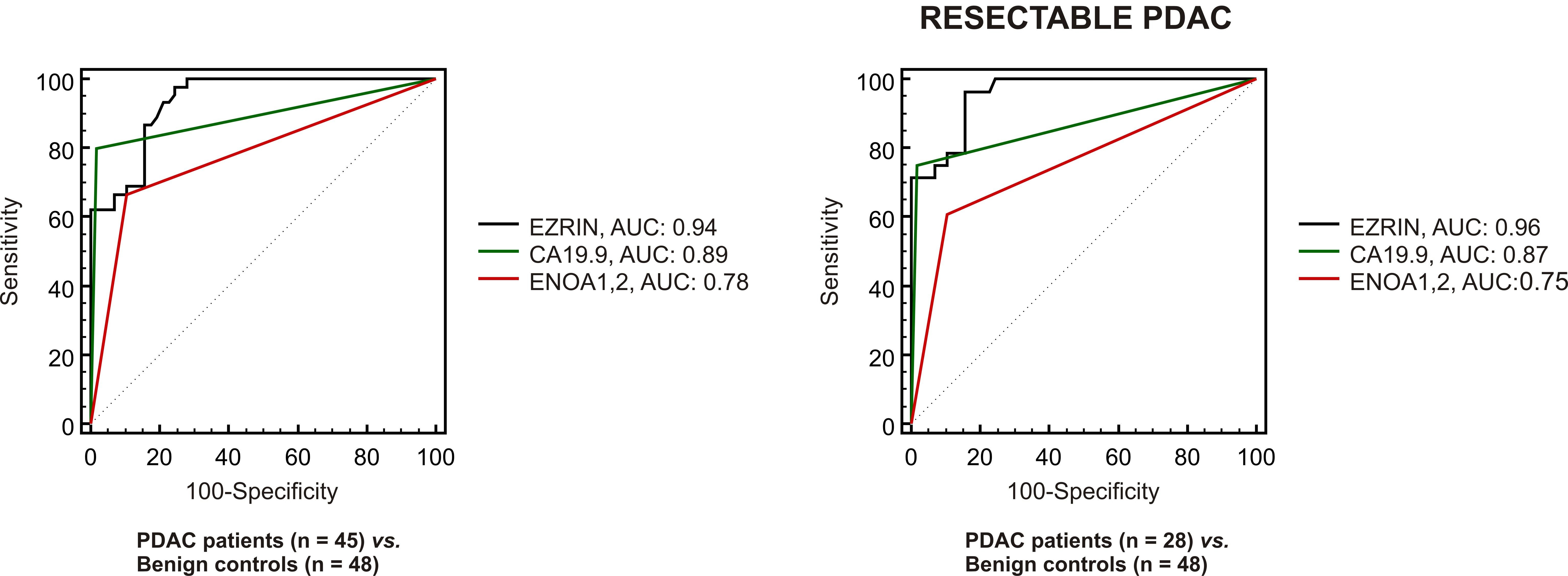
**

**Figure S4** ROC analysis of individually evaluated EZR-autoantibody, dichotomized CA19.9 serum level and ENOA1,2-autoantibody.
